# BgN-Score and BsN-Score: Bagging and boosting based ensemble neural networks scoring functions for accurate binding affinity prediction of protein-ligand complexes

**DOI:** 10.1186/1471-2105-16-S4-S8

**Published:** 2015-02-23

**Authors:** Hossam M Ashtawy, Nihar R Mahapatra

**Affiliations:** 1 Department of Electrical and Computer Engineering, Michigan State University, East Lansing, Michigan 48824, USA

**Keywords:** artificial neural networks, bagging, boosting, drug discovery, ensemble learning, protein-ligand binding affinity, scoring function, scoring power, virtual screening

## Abstract

**Background:**

Accurately predicting the binding affinities of large sets of protein-ligand complexes is a key challenge in computational biomolecular science, with applications in drug discovery, chemical biology, and structural biology. Since a scoring function (SF) is used to score, rank, and identify drug leads, the fidelity with which it predicts the affinity of a ligand candidate for a protein's binding site has a significant bearing on the accuracy of virtual screening. Despite intense efforts in developing conventional SFs, which are either force-field based, knowledge-based, or empirical, their limited predictive power has been a major roadblock toward cost-effective drug discovery. Therefore, in this work, we present novel SFs employing a large ensemble of neural networks (NN) in conjunction with a diverse set of physicochemical and geometrical features characterizing protein-ligand complexes to predict binding affinity.

**Results:**

We assess the scoring accuracies of two new ensemble NN SFs based on bagging (BgN-Score) and boosting (BsN-Score), as well as those of conventional SFs in the context of the 2007 PDBbind benchmark that encompasses a diverse set of high-quality protein families. We find that BgN-Score and BsN-Score have more than 25% better Pearson's correlation coefficient (0.804 and 0.816 vs. 0.644) between predicted and measured binding affinities compared to that achieved by a state-of-the-art conventional SF. In addition, these ensemble NN SFs are also at least 19% more accurate (0.804 and 0.816 vs. 0.675) than SFs based on a single neural network that has been traditionally used in drug discovery applications. We further find that ensemble models based on NNs surpass SFs based on the decision-tree ensemble technique Random Forests.

**Conclusions:**

Ensemble neural networks SFs, BgN-Score and BsN-Score, are the most accurate in predicting binding affinity of protein-ligand complexes among the considered SFs. Moreover, their accuracies are even higher when they are used to predict binding affinities of protein-ligand complexes that are related to their training sets.

## Background

Protein-ligand binding is essential for important physiological processes, such as cellular signaling, respiration, metabolism, defense against antigens, neuronal excitation and inhibition, hormone regulation, protein translation, etc., and so plays a fundamental role in drug design. To develop a new drug, first, a critical protein is identified in the pathway of a disease of interest. Then, small drug-like molecules called *ligands *are found or designed that will bind to the target protein, modulate its activity, and thus provide therapeutic benefit to the patient. The strength of binding of these drug-like molecules to the target protein is referred to as *binding affinity *and is commonly characterized using the dissociation constant between the ligand and its target macromolecule. *In vitro *determination of binding affinity is a time consuming and laborious task, especially for a large number of ligands. Due to prohibitive costs and delays associated with experimental drug discovery, pharmaceutical and biotechnology companies rely on virtual screening using computational molecular docking [1-3]. Typically, this involves docking tens of thousands to millions of ligand candidates into a target protein receptor's binding site and using a suitable scoring function (SF) to evaluate the binding affinity of each candidate to identify the top candidates as drug leads, and then to perform lead optimization [2]; it is also used for target identification [4]. Relative ranking of large number of ligands can also be predicted using the calculated binding affinities. Besides drug discovery, the bioactive molecules thus identified can be used as chemical probes to investigate the biochemical role of a target of interest [5]. Molecular docking also has applications in many structural bioinformatics problems, such as protein structure [6] and function prediction [7]. It has become attractive because of the ever-increasing number of available receptor protein structures and putative ligand drug candidates in publicly-accessible databases, such as the Protein Data Bank (PDB) [8], PDBbind [9], Cambridge Structural Database (CSD) [10], and corporate repositories.

In this work, we will build scoring functions based on an ensemble of neural networks to accurately and quickly predict protein-ligand binding affinity.

## Related work

Existing scoring functions employed in commercial and free molecular docking software fall in one of three main categories: force-field-based [11], empirical [12], or knowledge-based [13] SFs. Many comparative studies have found that these types of SFs are not accurate enough for reliable and successful molecular docking and virtual screening. A recent study examined a total of 16 popular scoring functions in their ability to reproduce experimental binding affinities of 195 protein-ligand complexes that encompass 65 different protein families [14]. Although these SFs are employed in mainstream commercial and academic molecular docking tools, the best performing SF achieved only mediocre accuracy of less than 0.65 in terms of Pearson's correlation between its predictions and measured binding affinities (BAs). These findings are in agreement with an earlier work by Wang et al. in which a related benchmark and scoring functions were examined [15]. Several of the evaluated SFs were empirical models derived via fitting linear regression equations to training data, but none were based on nonlinear modeling approaches. Therefore, we recently proposed random forests (RF), boosted regression trees (BRT), support vector machines (SVM), *k*-nearest neighbors (*k*NN), and multivariate adaptive regression splines (MARS) nonlinear scoring functions and compared their ligand scoring and ranking performances against the sixteen conventional SFs considered by Cheng et al. on the same benchmark test sets [16,17]. Our ML SFs, especially RF and BRT that are based on an ensemble of decision trees, have shown substantial improvement in binding affinity prediction accuracy over all the sixteen traditional scoring models.

Artificial neural networks (ANNs) have been previously used in computational drug development, but they have mostly been applied in QSAR modeling problems or in predicting the biological activity of ligands (active or not) against a target protein [18-21]. Their application in predicting binding affinity has been very rare and only reported in small scale experiments in which just a handful of protein-ligand complexes were used for training and validation [22,23]. Neural networks' poor generalization performance for higher dimensional data is perhaps the main reason for their limited use in scoring protein-ligand complexes in commercial docking tools. In this work, we propose novel SFs based on an ensemble of neural networks to predict binding affinity of protein-ligand complexes characterized using large and diverse number of descriptors. We train and test our models on hundreds of high-quality protein-ligand complexes and compare their accuracies against conventional and state-of-the-art scoring functions. We show that our NN SFs are resilient to overfitting and generalize well even when predicting BAs of complexes characterized by a large number of features.

## Key contributions

Conventional empirical SFs rest on the hypothesis that a linear regression function is sufficiently capable of modeling protein-ligand binding affinity [12,24]. Instead of assuming a predetermined theoretical function that models the unknown relationship between different energetic terms and binding affinity, an accurate nonparametric machine-learning method inspired from statistical learning theory is introduced in this work. We utilize a variety of features to build SFs *BgN-Score *and *BsN-Score *by combining a large number of diverse neural networks using bagging and boosting ensemble techniques, respectively. We show that BgN-Score and BsN-Score have scoring powers of 0.804 and 0.816 (in terms of Pearson's correlation coefficient), respectively, compared to 0.644 for the best conventional SF for a benchmark test set--this is a significant improvement in predictive power. In addition to this substantial 25% improvement, these ensemble NN SFs are also at least 19% (0.804 and 0.816 vs. 0.675) more accurate than SFs based on a single neural network. We also compare our proposed models to SFs based on random forests [25]. We found that our ensemble NN SFs surpass RF SFs (0.804 and 0.816 vs. 0.801)--the RF SFs we compare against are better than the RF SFs presented in the past in [16,26] because of the use of greater variety and number of features. Although NN and RF ensemble approaches are competitive with each other, the significance of NN ensemble SFs introduced in this work is two-fold. First, they represent a way to overcome the overfitting limitations of single neural network models that have been used traditionally in drug-discovery applications [18,19,21]. Second, neural networks have the ability to approximate any underlying function smoothly [27-29] in contrast to decision trees that model functions with step changes across decision boundaries [30].

We seek to advance structure-based drug design by designing SFs that significantly improve upon the protein-ligand binding affinity prediction accuracy of conventional SFs. Our approach is to couple the modeling power of ensemble learning algorithms with training datasets comprising hundreds of protein-ligand complexes with known high-resolution 3D crystal structures and experimentally-determined binding affinities and a variety of features characterizing the complexes. We will compare the predictive accuracies of BgN-Score, BsN-Score, single NN SF, RF SF, and existing conventional SFs of all three types, force-field, empirical, and knowledge-based, on diverse and homogeneous sets of protein families.

The remainder of the paper is organized as follows. The next section describes the protein-ligand complex database used for the comparative assessment of SFs, the physicochemical features extracted to characterize the complexes, the training and test datasets used, and the proposed and conventional SFs that we study. Next, we present results comparing the scoring powers of various SFs on diverse and homogeneous test sets of protein families. Finally, we summarize these results and conclude our work.

## Materials and methods

### Protein-ligand complex database

We used the same complex database that Cheng et al. used as a benchmark in their recent comparative assessment of sixteen popular SFs [14]. They obtained a *refined set *containing high-quality 3D structures of 1300 protein-ligand complexes from the 2007 version of PDBbind [9]. From this set, the curators of PDBbind built a test set that encompasses 65 different protein families, each of which binds to three different ligands to form a set of 195 unique protein-ligand complexes. This is called the *core set *and is mainly intended to be used for benchmarking docking and scoring systems. In order to be consistent with the comparative framework used to assess SFs in [14], we too consider the 2007 version of PDBbind. We use the core set as a test set in this work and denote it by *Cr*. A *primary training set*, denoted by *Pr*, was formed by removing all *Cr *complexes from the total 1300 complexes in the refined set of PDBbind. As a result, *Pr *contains 1105 complexes that are completely disjoint from *Cr *complexes.

### Protein-ligand complex characterization

For each protein-ligand complex, we extracted physicochemical features used in the empirical SFs X-Score [12] (a set of 6 features denoted by *X*) and AffiScore [31] (a set of 30 features denoted by *A*) and calculated by GOLD [32] (a set of 14 features denoted by *G*), and geometrical features used in the ML SF RF-Score [26] (a 36-feature set denoted by *R*). The software packages that calculate X-Score, AffiScore (from SLIDE), and RF-Score features were available to us in an open-source form from their authors and a full list of these features is provided in the appendix of [17]. The GOLD docking suite provides a utility that calculates a set of general descriptors for both molecules as separate entities and in a complex form. The full set of these features can be easily accessed and calculated via the *Descriptors *menu in GOLD. By considering all fifteen combinations of these four types of features (i.e., *X*, *A*, *R*, *G*, *X *∪ *A*, *X *∪ *R*, *X *∪ *G*, *A *∪ *R*, *A *∪ *G*, *R *∪ *G*, *X *∪ *A *∪ *R*, *X *∪ *A *∪ *G*, *X *∪ *R *∪ *G*, *A *∪ *R *∪ *G*, and *X *∪ *A *∪ *R *∪ *G*), we generated 15 versions of the *Pr *and *Cr *data sets, which we distinguish by using apropriate subscripts identifying the features used. For instance, *Pr_XR _*denotes the version of *Pr *comprising the set of features *X *∪ *R *(referred to simply as XR) and experimentally-determined binding affinity data for complexes in the *Pr *dataset.

### Artificial neural networks

**Figure 1:**
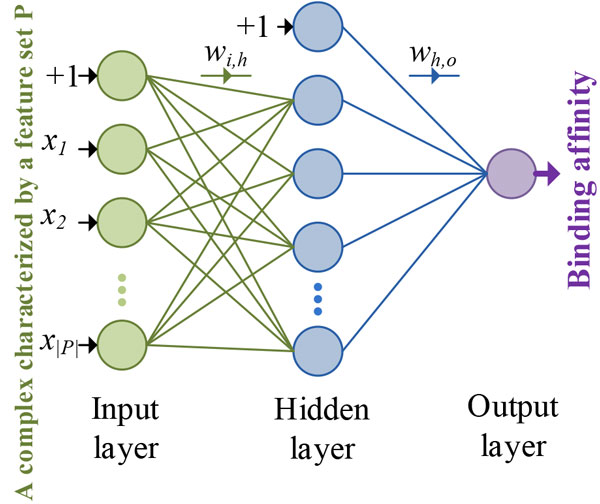
**Multi-layered perceptron, feed-forward neural network used to predict the binding affinity of a protein-ligand complex characterized by a set of features**. This model represents SNN-Score, the single neural network scoring function we build.

Computational methodologies inspired by networks of biological neurons, Artificial Neural Networks (ANNs), are employed in this work. Neural networks (NNs) have been applied in several drug design applications for both regression and classification problems [18,19,21]. Our ensemble approaches are based on feed-forward-back-propagation (FFBP) neural networks implemented in the R language package *nnet *[33]. Neural networks we fit using *nnet *are composed of an input layer that contains neurons corresponding to features extracted for complexes, an arbitrary number of neurons (20 in our experiments) in the hidden layer, and an output neuron for the output layer. These neurons are interconnected via weighted links as shown in Figure 1. The outputs of the input neurons are directed to all the neurons in the hidden layer. The outputs of the hidden layer neurons are also directed forward to the output neuron. The output of a network is calculated at its output neuron according to the formula:

(1) $$\hat{y}=f\left(x^{P}\right)=O\left(\overset{H}{\underset{h=0}{\sum }}\left(w_{h,o}S\left(\overset{\left|P\right|}{\underset{i=0}{\sum }}\left(w_{i,h}x_{i}\right)\right)\right)\right),$$

where $x^{P}\in {ℜ}^{\left|P\right|}$ is a feature vector representing a protein-ligand complex characterized by a feature set *P*, *f*(**x***^P^*) is the function that maps it to binding affinity $\hat{y}$, *O *is the activation function of the output neuron (linear in our case and defined simply as *O*(*u*) = *u*), *H *+ 1 is the total number of hidden neurons, *S *is the activation function for the hidden-layer neurons (logistic sigmoid in this work and expressed as *S*(*u*) = *e^u^*/(1 + *e^u^*)), *w_h,o _*refers to the weights associated with the links connecting the hidden to the output layer, *w_i,h _*represents the weights of input-to-hidden layer links, and *x_i _*is the *i^th ^*feature characterizing the protein-ligand complex. It should be noted that the weight variables *w*_0,*h *_in the *w_i,h _*set of weights serve as bias parameters and they are associated with an internal input variable *x*_0 _whose value is always fixed at one (*x*_0 _= 1). We similarly followed the same approach to absorb the bias parameter *w*_0,*o *_into the hidden layer set of weights *w_h,o _*by making the sigmoid function in Equation 1 output the value one (*S*(.) = 1) when *h *= 0. This topology is shown in Figure 1 where the value 1 is fed directly to the top neurons of the network's input and hidden layers. The network weights *w_i,h _*and *w_h,o _*are optimized such that they minimize the fitting criterion *E *defined as:

(2) $$E=\overset{N}{\underset{n=1}{\sum }}{\left(y_{n}-{\hat{y}}_{n}\right)}^{2}+\lambda \underset{\forall \;i,j}{\sum }w_{i,j}^{2},$$

where *N *is the number of protein-ligand complexes in the training data, *y_n _*and ${\hat{y}}_{n}$ are the measured and predicted binding affinities of the *n^th ^*complex, respectively, and *λ *is a regularization parameter. The parameter *λ *is also known as the weight decay and it guards against weights converging to large values. Introducing the weight decay parameter avoids the scenario of saturation at the output of the hidden-layer neurons. We scaled the input features to the range [0, 1] to effectively optimize the weights when regularization is considered. The accuracy of the network is maximized by performing thousands of randomized training rounds (3000 epochs) while imposing the regularization constraint on the weights.

### Limitations of ANN models and our approach to tackling them

Although multi-layer ANN models can theoretically approximate any nonlinear continuous function, their application in drug-discovery related problems has always been complicated by several challenges. Bioinformatics and cheminformatics data are typically high-dimensional. Since ANN models cannot handle large number of features efficiently, a pre-processing step prior to fitting the data using an ANN model is usually necessary. Feature subset selection using evolutionary algorithms or dimension reduction using, say, principal component analysis (PCA), is commonly applied to overcome this problem. However, valuable experimental information may be discarded when only a small subset of features is selected to build a prediction model. The dimensionality-reduction approach is also complicated by the fact that the underlying data distribution is unknown and hence making the right choice of which dimensionality-reduction technique to apply is a tricky problem in itself. In addition to these pre-processing issues, training ANN models is also a challenging task because their weights can not be guaranteed to converge to optimal values. This causes NN models to suffer from high variance errors which translate to unreliable SFs.

The aforementioned problems can be avoided and state-of-the-art performance can be achieved by combining predictions of hundreds of diverse and nonlinear NN models. We propose here ensemble methods based on ANNs. The ensemble itself is trained on all the features, although each network in the ensemble is fitted to only a subset of the features. This approach relieves us from carrying out feature subset selection or dimensionality reduction prior to training. In fact, the performance of the ensemble can even be improved by describing the data with more relevant features. Moreover, it is no longer critical to tune the weights of each network in the ensemble to optimal values as it is the case for a single NN model. Suboptimal weight tuning of individual networks could contribute to decreasing the inter-correlation between them, which translates to a diverse ensemble and therefore a more accurate model [25].

Our proposed NN ensemble models are inspired from the Random Forests [25] and Boosted Regression Trees [34] techniques in the formation of the ensembles. So far, the focus in ensemble learning has been more or less biased toward using decision trees as base learners in forming ensembles. Choosing trees as base learners is mainly due to their high flexibility and variance (low stability). High variance decreases inter-correlation between trees and therefore increases the overall ensemble model's accuracy. Instead of using decision trees as base learners, we employ here multi-layered perceptron (MLP) ANNs. ANN shares several characteristics with prediction trees. They are nonparametric, nonlinear, and have high variance. Moreover, both techniques are very fast in prediction. ANNs such as MLP, however, have the ability of modeling any arbitrary boundary smoothly while decision trees can only learn rectangular-shaped boundaries. Decision trees are typically pruned after training to avoid overfitting, whereas ANN uses regularization while the network weights are optimized during learning. We next describe our two new ensemble NN models.

### BgN-Score: ensemble neural networks through bagging

**Figure 2:**
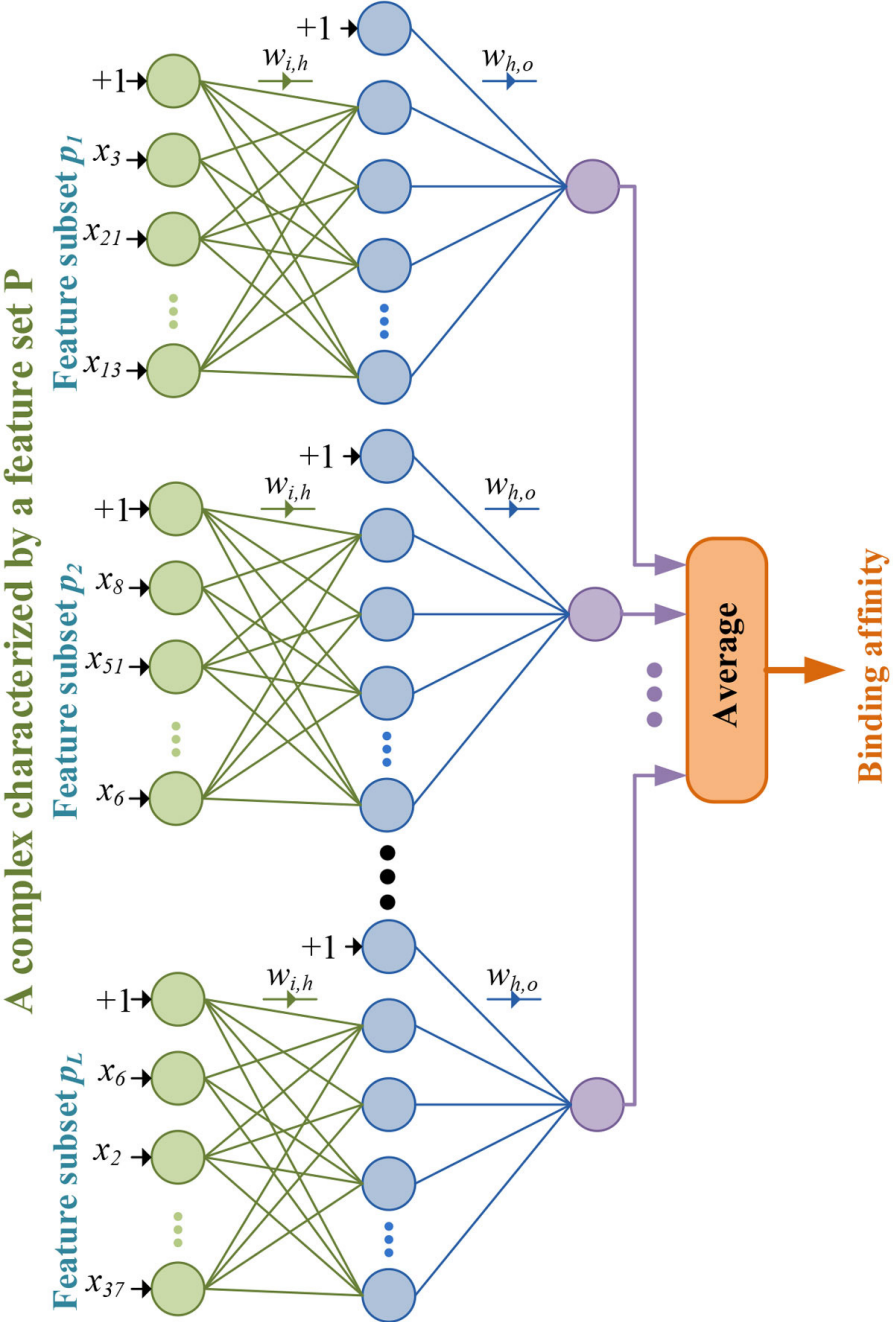
**BgN-Score: ensemble neural network SF using bagging approach**.

Bootstrap aggregation, or bagging for short, is a popular approach to construct an ensemble learning model. As the name implies and as indicated in the third step of Algorithm 1, the ensemble is composed of neural networks that are fitted to bootstrap samples from the training data. To further increase the diversity of the ensemble and decrease its training time, the inputs to each network *l *are a random subset (*p_l_*) of the total *P *features extracted for every protein-ligand complex (see Step 4). Feature sampling has proven effective in building tree-based ensemble algorithms such as Random Forests [25]. When the task is to predict the binding affinity of a new protein-ligand complex, the output is the aggregated average of the predictions of the comprising individual networks as shown in Algorithm 1 and depicted in Figure 2. This mechanism can be formally expressed as:

(3) $$\hat{y}=f\left(x^{P}\right)=\frac{1}{L}\overset{L}{\underset{l=1}{\sum }}f_{l}\left(x^{p_{l}}\right)=\frac{1}{L}\overset{L}{\underset{l=1}{\sum }}{\hat{y}}_{l},$$

where $x^{P}\in {ℜ}^{\left|P\right|}$ is a feature vector representing a protein-ligand complex characterized by a feature set *P*, *f*(**x***^P^*) is the function that maps it to binding affinity $\hat{y}\in ℜ$, $x^{p_{l}}\in {ℜ}^{\left|p_{l}\right|}$ is the same complex but characterized by a random subset *p_l _*of features (|*p*_*l*_| < |*P*|), *L *is the number of networks in the ensemble, and ${\hat{y}}_{l}$ is the prediction of each network *l *in the ensemble which is calculated at the output neuron according to Equation 1. The final bagging-based ensemble SF is referred to as *BgN-Score*.

### BsN-Score: ensemble neural networks through boosting

**Figure 3:**
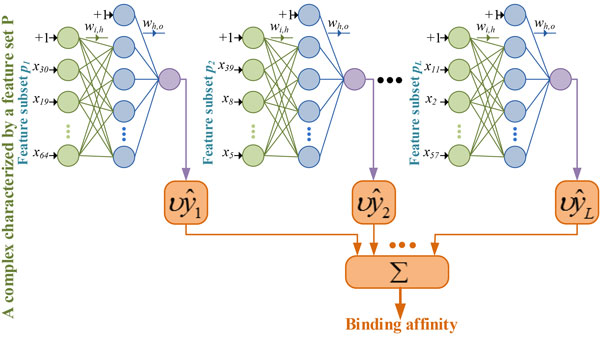
**BsN-Score: ensemble neural network SF using boosting approach**.

Boosting is an ensemble machine-learning technique based on a stage-wise fitting of base learners. The technique attempts to minimize the overall loss by boosting the complexes having highest predicted errors, i.e., by fitting NNs to (accumulated) residuals made by previous networks in the ensemble model. There are several different implementations of the boosting concept in the literature. The differences mainly arise from the employed loss functions and treatment of most erroneous predictions. Our proposed NN boosting algorithm in this work is a modified version of the boosting strategy developed by Cao et al. [35] and Friedman [34] in that we perform random feature subset sampling. This approach builds a stage-wise model as listed in Algorithm 2 and shown in Figure 3. The algorithm starts by fitting the first network to all training complexes. A small fraction (*ν *< 1) of the first network's predictions is used to calculate the first iteration of residuals $Y_{1}^{res}$ as shown in Step 3 of Algorithm 2. Step 3 also shows that the network *f*_1 _is the first term in the boosting additive model. In each subsequent stage *l*, a network is trained on a bootstrap sample of the training complexes described by a random subset *p_l _*of features (Steps 5 and 6). The values of the dependent variable of the training data for the network *l *are the current residuals corresponding to the sampled protein ligand complexes. The residuals for a network at each stage are the differences between previous stage residuals and a small fraction of its predictions. This fraction is controlled by the shrinkage parameter *ν *< 1 to avoid any overfitting. Network generation continues as long as the number of networks does not exceed a predefined limit *L*. Each network joins the ensemble with a shrunk version of itself. In our experiments, we fixed the shrinkage parameter to 0.001 which gave the lowest out-of-sample error. We refer to this boosting-based ensemble SF as *BsN-Score*.

### Neural networks and Random Forests scoring functions

In order to investigate the effectiveness of ensemble NN SFs in comparison to traditional NN models and ensemble decision-tree models, we trained and tested BgN-Score, BsN-Score, a single neural network SF referred to as *SNN-Score*, and a Random Forests (RF) SF on the *Pr *and *Cr *datasets, respectively, characterized by all fifteen combinations of the X, A, R, and G features discussed above. For a fair comparison of their potential, the parameters of these SFs were tuned in a consistent manner to optimize the mean-squared prediction errors on validation complexes sampled without replacement from the training set and independent of the test data. Out-of-bag instances were used as validation complexes for BgN-Score and RF, while a ten-fold cross-validation was conducted for BsN-Score and SNN-Score SFs. Out-of-bag (OOB) refers to complexes that are not sampled from the training set when bootstrap sets are drawn to fit individual NNs in BgN-Score models or decision trees in RF--on average, about 34% of the training set complexes are left out (or "out-of-bag") when bootstrap sets are drawn. The parameters that are tuned and their optimized values are as follows. (1) *L*: the number of base learners (neural networks in ensemble NN SFs and decision trees in RF) was set to 3000. (2) |*p*|: the size of the feature subset *p *randomly selected from the overall set of features *P *while constructing each neural network in ensemble NN SFs or the size of the randomly-selected feature subset used at each node of a decision tree to perform a binary split on the "best" feature in RF SF. This was set to 10 for ensemble SFs, except in the case where ensemble SFs are fitted to the 6 X-Score features when it was set to 3. The number of input neurons for SNN-Score is set to one more than the number of features used to characterize training and test complexes. All NN SFs have one output neuron per network that produces the binding affinity score. (3) *H *+ 1: the number of hidden-layer neurons in NN SFs was set to 20. (4) A total of 3000 training epochs and a decay value (*λ*) of 0.005 were used to optimize the weights for each network in the ensemble and single NN SFs. The training process of a network is terminated earlier if the fitting criterion defined in Equation 2 falls below 0.0001 before the maximum number of training epochs is completed. This threshold is the default value for the *abstol *parameter in the *nnet *package that we use. (5) *ν*: the shrinkage parameter for BsN-Score models was set to 0.001. (6) The weights of each network were randomly initialized in the range [-0.7,0.7]. The bounds of this uniform distribution are the default values for the *rang *parameter in the *nnet *package.

**Algorithm 1 **Algorithm for building BgN-Score: an ensemble NN SF using bagging

1: *Input: *training data **D **= {**X***^P^*, **Y**}, where $X^{P}=\left\{x_{1}^{P},\ldots ,x_{N}^{P}\right\}$, **Y **= {*y*_1_,...,*y_N_*}, and *N *is the number of training complexes.

2: **for ***l *= 1 to *L ***do**

3:   Draw a bootstrap sample $X_{l}^{P}$ from **X**^*P*^.

4:   Describe the complexes in the bootstrap sample $X_{l}^{P}$ using a random subset *p_l _*of features: $X_{l}^{P_{l}}$.

5:   From **Y**, draw the measured binding affinities of the complexes in the sample $X_{l}^{P}$ : **Y**_*l*_.

6:   Construct a new training set:$D_{l}=\left\{X_{l}^{p_{l}},Y_{l}\right\}$.

7:   Learn the current binding affinities by training an FFBP NN model *f_l _*on **D***_l_*.

8: **end for**

9: The final prediction of a protein-ligand complex **x***^P ^*is: $\hat{y}=f\left(x^{P}\right)=\frac{1}{L}\sum_{l=1}^{L}f_{l}\left(x^{p_{l}}\right)=\frac{1}{L}\sum_{l=1}^{L}{\hat{y}}_{l}$

**Algorithm 2 **Algorithm for building BsN-Score: an ensemble NN SF using boosting

1: *Input: *training data **D **= {**X***^P^*, **Y**}, where $X^{P}=\left\{x_{1}^{P},\ldots ,x_{N}^{P}\right\}$, **Y **= {*y*_1_,...,*y_N_*}, and *N *is the number of training complexes.

2: Construct {$D_{1}=\left\{X^{p_{1}},Y\right\}$ from **X***^P ^*by selecting a random subset *p*_1 _of features.

3: Train an FFBP NN model *f*_1 _on **D**_1 _and use it to predict BAs (${\hat{Y}}_{1}$) of the complexes $X^{p_{1}}$ . Calculate the residuals: $Y_{1}^{res}=Y-v{\hat{Y}}_{1}$.

4: **for ***l *= 2 to *L ***do**

5:   Draw a bootstrap sample $X_{l}^{P}$ from **X**^*P*^.

6:   Describe the complexes in the bootstrap sample $X_{l}^{P}$ using a random subset *p_l _*of features: $X_{l}^{P_{l}}$.

7:   From $Y_{l-1}^{res}$, draw the residuals corresponding to the complexes in the sample $X_{l}^{P}:Y_{l-1}^{res*}$.

8:   Construct a new training set: $D_{l}=\left\{X_{l}^{P_{l}},Y_{l-1}^{res*}\right\}$.

9:   Learn the current residuals by training an FFBP NN model *f_l _*on **D***_l_*.

10:   Calculate the predictions ${\hat{Y}}_{1}$ of the NN model *f_l _*on all $X^{p_{l}}$ training complexes in the original training set **D**.

11:   Update the residuals: $Y_{1}^{res}=Y_{l-1}^{res}-v{\hat{Y}}_{l}$

12: **end for**

13: The final prediction of a protein-ligand complex **x***^P ^*is: $\hat{y}=f\left(x^{P}\right)=\sum_{l=1}^{L}vf_{l}\left(x^{p_{l}}\right)=\sum_{l=1}^{L}v{\hat{y}}_{l}$

We distinguish the various NN models we built from each other using the notation *NN model::tools used to calculate features*. For instance, BsN-Score::XA implies that the SF is a boosted ensemble neural networks model that is trained and tested on complex sets described by *XA *features. For brevity, for each of SNN-Score, BgN-Score, BsN-Score, and RF models, we report results only for the feature combination (out of the fifteen possible) that yields the best performance on the validation complexes sampled without replacement from the training data and independent of the test set.

### Scoring functions under comparative assessment

We compare the scoring performance of our proposed NN models to those for sixteen scoring functions used in popular molecular docking software. The scoring accuracies of these sixteen SFs were computed by Cheng et al. in a recent study on the same benchmark we consider. The functions are listed in Table 1 which includes five SFs implemented in Discovery Studio, five SFs in SYBYL, three SFs in GOLD, one in Glide, and two standalone SFs. Nine of these SFs are empirical, four are knowledge-based, and the remaining three are based on force fields.

**Table 1:** The 16 conventional scoring functions and the molecular docking software in which they are implemented

Scoring function (SF)	Software	Type of SF	Reference
Jain	Discovery Studio	Empirical	[37]
LigScore		Knowledge based	[38]
Ludi		Empirical	[39]
PLP		Empirical	[40]
PMF		Knowledge based	[41]
ChemScore	SYBYL	Empirical	[24]
D-Score		Force-field based	[11]
G-Score		Force-field based	[32]
F-Score		Empirical	[42]
PMF-Score^1^		Knowledge based	[41]
ASP	GOLD	Empirical	[43]
ChemScore^2^		Empirical	[24]
GoldScore^3^		Force-field based	[32]
GlideScore	Glide	Empirical	[44]
DrugScore	*Standalone*	Knowledge based	[45]
X-Score	*Standalone*	Empirical	[12]

^1 ^SYBYL's implementation of PMF

^2 ^GOLD's implementation of ChemScore

^3 ^GOLD's implementation of G-Score

Some of the scoring functions have several options or versions, these include DrugScore, LigScore, LUDI, PLP, and X-Score. For conciseness, we only select the version that has the highest scoring accuracy on the PDBbind benchmark that was considered by Cheng et al. [14]. Our NN model selection, however, was based on the validation complexes sampled without replacement from the training data which is independent of the test set. Therefore, the gap in performance between our proposed SFs and the conventional models we report in the following sections could in fact be even bigger if model/version selection of conventional SFs was done based on their performance on independent validation sets instead of the test set *Cr*.

## Results and discussion

### Evaluation of scoring functions

Scoring power of SFs quantifies their ability to accurately predict protein-ligand binding affinity or reproduce it for complexes with known experimental BA data. The similarity between the predicted and measured BAs are calculated using Pearson's (*R_p_*) and Spearman's (*R_s_*) correlation coefficients, the standard deviation (SD) of errors, and the root-mean square-error (RMSE). Pearson's correlation coefficient measures the linear relationship between two variables as follows:

$$R_{p}=\frac{\sum_{i=1}^{N}\left[({\hat{Y}}_{i}-\bar{\hat{Y}})(Y_{i}-\bar{Y})\right]}{\sqrt{\sum_{i=1}^{N}{\left({\hat{Y}}_{i}-\bar{\hat{Y}}\right)}^{2}\sum_{i=1}^{N}{\left(Y_{i}-\bar{Y}\right)}^{2}}},$$

where *N *is the number of complexes and ${\hat{Y}}_{i}$ and *Y_i _*are the predicted and measured binding affinities of the *i*th complex, respectively. The average values of the predicted and experimentally measured affinities for all complexes are $\bar{\hat{Y}}$ and $\bar{Y}$, respectively. Spearman's correlation coefficient is used to evaluate the correlation between the predicted and measured BAs in terms of their ranks and it is defined as follows:

$$R_{s}=1-\frac{6\sum_{i=1}^{N}d_{i}^{2}}{N(N^{2}-1)},$$

where *d_i _*is the difference in ranks of the predicted and measured affinities of the *i*th complex.

The SF that achieves the highest correlation coefficient (maximum is one) for some dataset is considered more accurate than its counterparts that realize smaller *R_p _*and/or *R_s _*values (minimum is negative one). Another measure of scoring power we report here is the standard deviation (SD) of errors between predicted and measured BAs (in − log *K_i _*or − log *K_d _*units). To calculate this statistic for a given SF, a linear model that correlates predicted scores $\hat{Y}$ to the measured ones *Y *is first evaluated: $Y=\beta_{0}+\beta_{1}\hat{Y}$, where *β*_0 _and *β*_1 _are the intercept and the slope of the model, respectively. The SD statistic can then be computed as follows [15]:

$$\text{SD}=\sqrt{\frac{\sum_{i=1}^{N}{\left(Y_{i}-(\beta_{0}+\beta_{1}{\hat{Y}}_{i})\right)}^{2}}{N-2}}.$$

The root-mean square-error (RMSE) of the predicted scores is calculated as:

$$\text{RMSE}=\sqrt{\frac{\sum_{i=1}^{N}{\left(Y_{i}-{\hat{Y}}_{i}\right)}^{2}}{N}}.$$

SFs that yield smaller SD and RMSE values usually realize higher *R_p _*and *R_s _*values, and therefore have higher scoring power than models with large SD and RMSE statistics.

### Ensemble neural networks vs. other approaches on a diverse test set

We trained three neural network SFs (SNN-Score, BgN-Score, and BsN-Score) and an RF scoring model on the primary training set *Pr *and evaluated their scoring performance on an independent test set of 195 diverse protein-ligand complexes from 65 different protein families.

Table 2 lists the scoring powers of these models and the same performance statistics of the sixteen SFs collected by Cheng et al. on the same test set. We also report the scoring performances of NN and RF SFs on the training set *Pr *by using out-of-sample validation to show how close the predicted BAs are to the experimentally-measured ones in terms of RMSE. Therefore, this statistic indicates whether SFs deemed accurate on training data will also be reliable scoring models on the test set *Cr*. This measure was not calculated for the conventional SFs (except X-Score) since we do not have access to their training-set BA values. All the scoring power metrics (i.e., *R*_p_, *R*_s_, SD, and RMSE) indicate that our proposed SFs and RF are the most accurate in predicting the binding affinities of the independent test set complexes and out-of-sample training data. BsN-Score outperforms the most accurate conventional SF, X-Score::HMScore, by at least 26% in terms of Pearson's correlation coefficients, which is 0.816 and 0.644 for both SFs, respectively. BgN-Score also achieves excellent performance of 0.804 which is about 25% improvement over X-Score::HMScore.

**Table 2:** Comparison of the scoring powers of BsN-Score, BgN-Score, SNN-Score, Random Forests (RF), and 16 conventional SFs on the core test set *Cr*

Scoring function	*N* ^1^	*R_p_* ^2^	*R_s_* ^3^	*SD* ^4^	RMSE_test_^5^	RMSE_train_^6^
BsN-Score::XARG	195	0.816	0.799	1.38	1.386	1.366
BgN-Score::XARG	195	0.804	0.798	1.42	1.449	1.403
RF::XARG	195	0.801	0.790	1.43	1.498	1.442
SNN-Score::X	195	0.675	0.685	1.76	1.760	1.704
X-Score::HMScore	195	0.644	0.705	1.83	1.865	1.730
DrugScore^CSD^	195	0.569	0.627	1.96	-	-
SYBYL::ChemScore	195	0.555	0.585	1.98	-	-
DS::PLP1	195	0.545	0.588	2.00	-	-
GOLD::ASP	195	0.534	0.577	2.02	-	-
SYBYL::G-Score	195	0.492	0.536	2.08	-	-
DS::LUDI3	195	0.487	0.478	2.09	-	-
DS::LigScore2	193	0.464	0.507	2.12	-	-
GlidScore-XP	178	0.457	0.435	2.14	-	-
DS::PMF	193	0.445	0.448	2.14	-	-
GOLD::ChemScore	178	0.441	0.452	2.15	-	-
SYBYL::D-Score	195	0.392	0.447	2.19	-	-
DS::Jain	189	0.316	0.346	2.24	-	-
GOLD::GoldScore	169	0.295	0.322	2.29	-	-
SYBYL::PMF-Score	190	0.268	0.273	2.29	-	-
SYBYL::F-Score	185	0.216	0.243	2.35	-	-

^1 ^Number of complexes in *Cr *with positive (favorable) binding scores using this SF [14].

^2 ^*R_p _*is the Pearson correlation coefficient between predicted and measured BA values of complexes in *Cr*.

^3 ^*R_s _*is the Spearman correlation coefficient between predicted and measured BA values of complexes in *Cr*.

^4 ^SD is the standard deviation of errors between predicted and measured BA values of complexes in *Cr *based on Equation 3 in [15].

^5 ^RMSE is the root-mean-square of errors between predicted and measured BA values of the test complexes in *Cr*. Test RMSE is not available for conventional SFs except for X-Score::HMScore that we have re-constructed.

^6 ^RMSE is the root-mean-square of errors between predicted and measured BA values of out-of-sample complexes in the training set *Pr*. Training RMSE is not available for conventional SFs except for X-Score::HMScore that we have re-constructed.

There are two main reasons for the superior performance of ensemble SFs. First, more numerous and varied features more fully characterize protein-ligand interactions. Thus we find that BsN-Score and BgN-Score SFs employing all four features types considered (X, A, R, and G features) are more accurate than the same SFs employing fewer features. Second, and more important, the learning model of ensemble SFs is nonlinear and flexible and can exploit a large number of features while being resilient to overfitting. Thus we find that SNN-Score::X (for which *R_p _*= 0.675) is more accurate compared to the versions of SNN-Score employing one of A, R, or G features only as well as SNN-Score::XARG (for which *R_p _*= 0.517) because single neural network models overfit the training complexes when characterized by a large number of features. We attempted to decrease the effect of overfitting by conducting feature reduction using PCA which helped increase the performance of SNN-Score::XARG to *R_p _*= 0.667. However, the predictions of SNN-Score::XARG are still substantially less accurate than those of BsN-Score::XARG and BgN-Score::XARG even though the first 10 principle components we used to calculate the 10 new features explain more than 0.997 of the total variance in the raw XARG features. Further, the significance of the ensemble modeling approach can be gauged from the fact that even with a single type of feature, BsN-Score::A and BgN-Score::A yield accuracies of *R_p _*= 0.780 and 0.775, respectively, which are within *∼ *4% of the accuracies of BsN-Score::XARG and BgN-Score::XARG.

Table 2 also shows that the ensemble NNs SFs BsN-Score::XARG and BgN-Score::XARG are more accurate than the decision-trees-based ensemble SF RF::XARG, though the latter comes a close third (0.816 and 0.804 vs. 0.801); note that RF::XARG is considered here since it was found to have superior accuracy compared to RF::R presented in [26] and RF::A presented in [16]. We believe this difference in performance, although small, is mainly attributable to the way the base learners of these ensemble models approximate the unknown function. Decision trees model the unknown function by partitioning training data into smaller subsets from which a prediction is calculated. Such a procedure creates a series of non-overlapping regions with axis-parallel decision boundaries. The numerical values associated with each region are typically the average BA of the training data subset belonging to that partition which could be significantly different from the neighboring regions. This could create a rough and abrupt approximation of the unknown function. On the other hand, NNs with hidden units can closely and smoothly model any nonlinear continuous function. In addition, hidden neurons may create new important features that would otherwise be impossible to extract directly from protein-ligand complexes. These two factors minimize the bias error of NN models, but may lead to increased variance or instability as in the case of single neural network SFs. The proposed boosting and bagging ensemble learning approaches greatly reduce the variance error. Such simultaneous reduction in bias and variance errors makes the ensemble NN SFs the most accurate BA predictors compared to the other 18 scoring functions listed in Table 2.

### Ensemble neural networks vs. other approaches on homogeneous test sets

It has been observed that around 92% of existing drug targets are similar to proteins already present in the Protein Data Bank, which is the primary source of our training and validation complexes [36]. Based on this finding and the similar overlap relationship between training and test set proteins in the previous experiment, we believe that the scoring performance of the SFs listed in Table 2 should be expected in typical molecular docking and virtual screening campaigns. For each protein family in that experiment's test set, there is at least one protein family in the training set of our proposed NN and RF SFs, but the two sets share no protein-ligand complexes when these pairs of compounds are considered as whole biological units. We describe here a more stringent experiment to assess the generalization of the NN and RF SFs when they are applied to score ligands for novel drug targets. In this experiment, we evaluate the BA predictive accuracy of the NN SFs on four protein families not present in their training set. These protein families are the most frequent in our data and include 112 HIV protease, 73 trypsin, 44 carbonic anhydrase, and 38 thrombin complexes. A test set for each of these protein families was constructed by sampling all complexes formed by that protein from the training (*Pr *) and the test (*Cr *) sets. The training complexes corresponding to each of these four test sets are the remaining protein-ligand pairs in *Pr*. For each protein family, we fitted the proposed NN and RF models to the corresponding independent training complexes and validated them on the test set complexes that are formed between that type of protein and a unique set of co-crystallized ligands. The prediction accuracy of our proposed models and the top four conventional scoring functions on complexes formed by the four protein types are shown in Table 3.

**Table 3:** Comparison of the scoring powers of BsN-Score, BgN-Score, SNN-Score, Random Forests (RF), and the four top performing conventional SFs on four protein-family-specific tests sets.

HIV protease (*N *= 112)						Trypsin (*N *= 73)					
	
Scoring function	*R_p_* ^1^	*R_s_* ^2^	*SD* ^3^	RMSE^4^	D^5^	Scoring function	*R_p_* ^1^	*R_s_* ^2^	*SD* ^3^	RMSE^4^	D^5^
X-Score::HPScore	0.341	0.339	1.54	1.509	N	SYBYL::ChemScore	0.829	0.773	0.95	-	U
BsN::XARG	0.290	0.230	1.56	1.705	Y	DS::Ludi2	0.823	0.791	0.96	-	U
RF::XARG	0.289	0.219	1.519	1.719	Y	X-Score::HSScore	0.817	0.824	0.97	1.401	N
BgN-Score::XARG	0.287	0.209	1.58	1.860	Y	DS::PLP2	0.797	0.774	1.02	-	U
SYBYL::ChemScore	0.255	0.228	1.58	-	U	BgN-Score::XAR	0.776	0.719	1.06	1.070	Y
DrugScore::PairSurf	0.225	0.170	1.59	-	U	RF::XAR	0.774	0.753	1.07	1.133	Y
DS::PMF04	0.183	0.200	1.61	-	U	BsN-Score::AR	0.766	0.709	1.08	1.119	Y
SNN-Score::X	0.039	0.048	1.64	2.255	Y	SNN-Score::X	0.735	0.672	1.14	1.209	Y

RF::XARG	0.964	0.975	0.44	0.588	N	BsN-Score::XARG	0.937	0.920	0.59	0.678	N
BsN-Score::XARG	0.918	0.922	0.64	0.710	N	RF::XARG	0.934	0.08	0.60	0.657	N
BgN-Score::XARG	0.848	0.808	1.02	1.024	N	BgN-Score::XARG	0.892	0.848	0.76	0.805	N
SNN-Score::X	0.748	0.716	1.08	1.085	N	SNN-Score::X	0.829	0.789	0.940	0.957	N

**Carbonic anhydrase (*N *= 44)**						**Thrombin (*N *= 38)**					
	
**Scoring function**	***R_p_* ^1^**	***R_s_* ^2^**	***SD* ^3^**	**RMSE^4^**	**D^5^**	**Scoring function**	***R_p_* ^1^**	***R_s_* ^2^**	***SD* ^3^**	**RMSE^4^**	**D^5^**

DS::PLP2	0.800	0.772	0.84	-	U	SNN-Score::X	0.756	0.704	1.38	1.433	Y
SYBYL::G-Score	0.706	0.646	0.99	-	U	BgN-Score::XARG	0.722	0.726	1.48	1.552	Y
SYBYL::ChemScore	0.699	0.631	1.00	-	U	BsN-Score::XARG	0.699	0.637	1.58	1.603	Y
BsN-Score::X	0.674	0.434	1.03	3.418	Y	RF::XARG	0.697	0.693	1.52	1.674	Y
SNN-Score::X	0.631	0.451	1.08	3.561	Y	DS::PLP1	0.667	0.672	1.58	-	U
SYBYL::PMF-Score	0.627	0.618	1.09	-	U	SYBYL::G-Score	0.667	0.626	1.58	-	U
BgN-Score::XA	0.625	0.423	1.09	3.642	Y	X-Score::HSScore	0.666	0.586	1.58	1.737	N
RF::XARG	0.601	0.374	1.11	3.393	Y	DrugScore::Pair	0.651	0.622	1.61	-	U

BsN-Score::XARG	0.948	0.921	0.44	1.004	N	BsN-Score::XARG	0.913	0.938	0.86	1.155	N
RF::XARG	0.910	0.860	0.57	1.140	N	RF::XARG	0.910	0.934	0.86	1.125	N
BgN-Score::XARG	0.884	0.766	0.65	1.320	N	BgN-Score::XARG	0.858	0.876	1.08	1.320	N
SNN-Score::X	0.652	0.310	1.05	1.687	N	SNN-Score::X	0.761	0.756	1.37	1.374	N

^1 ^*R_p _*is the Pearson correlation coefficient between predicted and measured BA values of complexes in this protein-family-specific test set.

^2 ^*R_s _*is the Spearman correlation coefficient between predicted and measured BA values of complexes in this protein-family-specific test set.

^3 ^SD is the standard deviation of errors between predicted and measured BA values of complexes in this protein-family-specific test set.

^4 ^RMSE is the root-mean-square of errors between predicted and measured BA values of the test complexes in in this protein-family-specific test set. Test RMSE is not available for conventional SFs except for X-Score that we have re-constructed. Training RMSE is not reported in this table because the values are very similar to RMSEtrain in Table 1 due to the overlap between the training data sets of the two experiments.

^5 ^This indicates whether the test set complexes are disjoint from (D = Y) or overlap with (D = N) the training set complexes for NN and RF models. Any overlap between the training and test data of the conventional SFs is unknown (D = U) to us.

Examining the upper portion of the table for the four families where the test and training sets are disjoint for the NN and RF SFs, we notice that the predictive accuracy of all SFs varies from poor to good depending on the protein family. All SFs have failed to reproduce the experimental binding affinities for the ligands that bind to HIV protease proteins. The highest Pearson's correlation value between predicted and true BAs is less than 0.35, which is the case for the scoring function X-Score::HPScore. Improper characterization of enthalpic and entropic forces for HIV protease complexes could be the main reason for these erroneous predictions [14]. The significant conformational changes observed during binding as well as the lack of similar proteins in the training set could also result in such inaccurate estimations for BA. The scoring accuracy on the other three protein families is substantially better. The binding affinities for ligands bound to trypsin were predicted with an accuracy of at least *R_p _*= 0.735. Discovery Studio's empirical SF PLP2 shows the highest accuracy on the carbonic anhydrase dataset with a linear correlation value of 0.800. The most accurate models on the thrombin test set are the NN SFs and RF with R_p _values of 0.697 and better, followed by the conventional scoring functions.

It can be observed that the SF based on a single NN, SNN-Score, performs relatively poorly overall, except in one case. In some of these test sets, a few conventional SFs perform better than the ensemble NN SFs. This behavior can be attributed to the possibility of some overlap between the training complexes of the conventional approaches and the four family-specific test sets. As discussed earlier, the protein families of training and test complexes for the NN and RF models do not overlap and they are completely disjoint. When we retrain ensemble NN and RF SFs on the original training set (*Pr *), which overlaps with the family-specific test sets, and assess their scoring power on the four homogeneous test sets, we notice that the predictions of the proposed SFs and RF are near perfect as listed in the lower portion of Table 3.

The results listed in Tables 2 and 3 show the performance of the proposed and conventional SFs on target proteins when they are partially or fully encountered in their training sets, or completely novel for them. Therefore, we believe that these results are very useful in estimating the accuracy of our scoring models given the number of solved structures of the drug target with other ligands and the availability of their binding data.

## Conclusion

Our experiments have shown that the proposed neural networks SFs, BsN-Score and BgN-Score, achieved the best results in reproducing experimental binding affinity for large and diverse number of protein-ligand complexes. We further found that ensemble models based on NNs surpass SFs based on the decision-tree ensemble technique Random Forests. SFs that were trained on a single neural network, which have traditionally been used in drug-discovery applications, showed linear correlation (*R_p_*) of only 0.675 between observed and predicted binding affinities. On the other hand, BsN-Score and BgN-Score along with RF-Score far outperform the best of existing conventional knowledge-based, force-field-based, and empirical SFs (*R_p _*= 0.816 and 0.804 vs. 0.644, respectively) and those based on a single neural network. The accuracies of ensemble NN SFs are even higher when they predict binding affinities for protein-ligand complexes that are related to their training sets. The high predictive accuracy of ensemble SFs BsN-Score and BgN-Score is due to the following three factors: (i) the low bias error of the highly-nonlinear neural network base learners, (ii) the low variance error achieved using bagging and boosting, and (iii) the employed diverse set of features we extract for protein-ligand complexes. We aim to improve the scoring powers of BsN-Score and BgN-Score even further in the future. We will periodically update the training data to include larger number of complexes with more protein families and ligands. We will analyze the effect of including more training complexes on the gain in predictive accuracy of NN SFs. We will also systemically examine their improvement patterns upon scoring ligands of specific protein families when complexes formed by those families have varying degrees of presence in the training data. Furthermore, we will develop new tools to extract a diverse and large number of physiochemical descriptors about the protein, the ligand, and the complex as a whole. We believe the suggested enhancement approaches will make the NN SFs even more useful for accurate molecular docking and scoring.

## Competing interests

The authors declare that they have no competing interests.

## Authors' contributions

Devised the comparison techniques and experiments: N.M. and H.A. Implemented the techniques and carried out the experiments: H.A. Analyzed the results: N.M. and H.A. Wrote the paper and revised it: H.A. and N.M.

## Declarations

The publication costs for this article were sourced from the National Science Foundation under Grant No. 1117900.

This article has been published as part of *BMC Bioinformatics *Volume 16 Supplement 4, 2015: Selected articles from the 9th IAPR conference on Pattern Recognition in Bioinformatics. The full contents of the supplement are available online at http://www.biomedcentral.com/bmcbioinformatics/supplements/16/S4.
